# Neurodevelopment of children exposed intra-uterus by Zika virus: A case series

**DOI:** 10.1371/journal.pone.0229434

**Published:** 2020-02-28

**Authors:** Pedro Massaroni Peçanha, Saint Clair Gomes Junior, Sheila Moura Pone, Marcos Vinicius da Silva Pone, Zilton Vasconcelos, Andrea Zin, Renata Hydee Hasue Vilibor, Roozemeria Pereira Costa, Maria Dalva Barbosa Baker Meio, Karin Nielsen-Saines, Patricia Brasil, Elizabeth Brickley, Maria Elisabeth Lopes Moreira

**Affiliations:** 1 Instituto Fernandes Figueira–Fiocruz, Rio de Janeiro, Brazil; 2 Universidade de São Paulo- USP, São Paulo, Brazil; 3 Universidade da California- UCLA, Los Angeles, California, United States of America; 4 Instituto Nacional de Infectologia-Fiocruz, Rio de Janeiro, Brazil; 5 London School of Hygiene & Tropical Medicine, London, England, United Kingdom; Arizona State University, UNITED STATES

## Abstract

The main goal of this manuscript was to investigate the neurodevelopment of children exposed by Zika virus in the intrauterine period who are asymptomatic at birth. Newborns with documented Zika virus exposure during the intrauterine period who were asymptomatic at birth were followed in the first two years of life for neurodevelopment using Bayley III test. Children were classified as having normal or delayed neurodevelopment for age based on most recent Bayley III evaluation results. Eighty-four infants were included in the study. The first Bayley III evaluation was performed at a mean chronological age of 9.7±3.1 month; 13 children (15%) had a delay in one of the three domains, distributed as follow: 10 (12%) in the language domain and 3 (3.5%) in the motor domain. The most recent Bayley III evaluation was performed at a mean age 15.3±3.1 months; 42 children (50%) had a delay in one of the three domains: 4 (5%) in cognition, 31 (37%) in language, and 20 (24%) in motor performance. There were no statistical differences in Gender, Gestational Age, Birth Weight and Head Circurference at birth between children with normal and delayed neurodevelopment for age. A very high proportion of children exposed ZIKV during pregnancy who were asymptomatic at birth demonstrated a delay in neurodevelopment, mainly in the language domain, the first two years of life.

## Introduction

The first 1,000 days of life are critical for children to reach their full development potential [1]. Insults occurring during pregnancy and the perinatal period can affect the brain development with deleterious consequences to future life, creating a range of impairments which can interfere with social capacity and function [2,3]. Adequate nutrition, psychosocial stimulation, and protection must be provided to children in this adverse setting [4].

Following the Zika Virus (ZIKV) epidemic in the Northeast of Brazil between 2014 and 2015, an increase in the number of newborn with severe microcephaly and other malformations were observed, characterizing the Congenital Zika Virus Syndrome (CZS) [5–8]. The potential for transmission of this infection to other countries and the severity of symptoms observed in infants of infected mothers led the World Health Organization (WHO) to declare that ZIKV infection was a Public Health Emergency of international concern. As such, the WHO recommended studies for better understanding ZIKV pathogenesis, vaccine development, as well as public health guidance for disease control and prevention [9,10].

Although, many newborns were affected by ZIKV exposure *in utero*, some were born asymptomatic, despite having proven intrauterine infection. It is well established that this ZIKV can causes microcephaly in newborns and is associated with Guillain-Barré Syndrome in adults [11–13]. However, it is still *unknown* whether this virus can cause an asymptomatic infection at birth, leading subsequently to impaired brain development. The consequences would be apparent later in childhood, depending on the functions compromised. The main objective of the present study was to investigate the neurodevelopment of newborns exposed with ZIKV in the intrauterine period, who were asymptomatic at birth, during the first two years of life.

## Material and methods

### Design, setting, and population

This is an exploratory case series in which newborns exposed by ZIKV in intrauterine period who were asymptomatic at birth were followed from birth to 2 years of life, between May 2016 and January 2018, in the outpatient clinic at Instituto Fernandes Figueira (IFF)-Fundação Oswaldo Cruz (Fiocruz), a reference center for congenital infection in Rio de Janeiro, Brazil.

#### Inclusion and exclusion criteria

We included infants whose mothers had Zika positive reverse-transcriptase polymerase chain reaction (RT-PCR) during pregnancy. Exposure by ZIKV was defined by the presence of at least one positive RT-PCR result from either the mother’s urine or blood, placenta, amniotic fluid or the infant’s blood, urine, or cerebrospinal fluid (CSF). They were considered exposed to ZIKV [11,13]. Newborns exposed by ZIKV in the intrauterine period who were asymptomatic at birth (i.e., normal physical and neurological exam in the first month of life according Dubowitz test [14] and normal brain ultrasound), who had at least two neurodevelopmental evaluations during the first two years of life were included.

Newborns with CZS, TORCH (Toxoplasma gondii, Treponema pallidum, rubella, cytomegalovirus, and herpes simplex), Varicella-zoster virus, Epstein-Barr virus, Human Immunodeficiency Virus and congenital anomalies, unrelated to ZIKV infection were excluded from the study. The definition criteria of CZS was the presence of at least one of the following findings: severe microcephaly with partially collapsed skull; thin cerebral cortices with subcortical calcifications; macular scarring and focal pigmentary retinal mottling; congenital contractures; and marked early hypertonia and symptoms of extrapyramidal involvement [8].

### Variables and data collection

- Social demographics: maternal age (years), paternal age (years), Tertiary mother’s level of education (more than 12 years in school) [15] and household income (minimum wage is US$ 288.40/month [16]);
- Maternal pregnancy parameters: trimester of Zika infection (1^st^, 2^nd^ or 3^rd^ trimester), presence of gestational hypertension, pre-eclampsia, alcohol or tobacco use during gestation, type of delivery (vaginal or cesarean section) and multiple gestations;
- Neonatal parameters: gestational age, gender, birth weight, birth weight Z-score, weight for gestational age using as reference the Intergrowth 21^st^ chart [17], head circumference (HC), HC Z-score, first and fifth minute Apgar score and use of mechanical ventilation;
- Post-natal follow-up: breastfeeding history until 6 months of age, hospital admissions during the first two years of life, Delta head circumference (Delta HC), neurological abnormalities (hypertonia, hypotonia, hyperreflexia, seizures, hyperexcitability, spasticity, irritability, tremors) and scores of Bayley III motor, cognitive and language domain scores [18].

Household income was classified into three ranges: up to 2 minimum wages, from 3 to 5 and above 5 minimum wages, considering values at the moment of evaluation. Gestational age was defined as the number of full weeks by the Ballard examination [19]. Weights were measured using a digital scale with a precision of 5g. The head circumference (cm) was measured with an inextensible tape. The Intergrowth 21^st^ and the WHO charts were used for the calculation of the Z-scores measurements at each point of evaluation [17,20].

The weight adequacy for gestational age was classified using the Intergrowth 21^st^ chart at birth. Newborns with a birthweight Z-score less than -2 standard deviation (SD) from the mean were considered small for gestation age (SGA), between -2 SD and 2 SD as appropriate for gestational age (AGA), and higher than 2 SD as large for gestational age (LGA). The Delta HC was obtained by the difference between the HC at the nearest visit for the Bayley assessment and the HC at birth.

The Development Scales for Toddlers and Infants Third Edition (Bayley III) was used [18]. This scale provided scores for three major development domains: motor, cognition, and language, varying from 40 to 160, with a mean of 100. Scores between 85 and 115 indicate normal development, while scores below 85 (-1 SD) indicate a developmental delay in the domain evaluated. At least two evaluations were performed by trained examiners between the ages of 6 and 24 months during the follow-up. The adjusted age was considered for the children who were born preterm.

The first and the last Bayley results available in each domain were compared to investigate the evolution of neurodevelopment. Results of the last Bayley evaluation were used to determine the child’s development in each domain. Children with scores in the normal range in all three domains were considered developmentally normal; children any score bellow 85 (-1 SD) in any of three domains were considered delayed.

#### Data record and analysis

All data were collected with standardized forms and stored using the software Microsoft Access. Categorical variables were analyzed as frequency and the numerical variables as mean and SD. Potential association were evaluated between neurodevelopment delay and all collected variables. Chi-square or Fisher exact text (for frequencies below 5) were used to evaluate the statistical association of the categorical variables. Student t-test (variables with a normal distribution) or the Mann-Whitney test (variables without a normal distribution) were used for the numerical variables.

#### Ethical issues

Study population was drawn from the cohort “Vertical exposure to Zika virus, and its consequences on children’s neurodevelopment” registered in ClinicalTrials.gov (NCT 03255369), and approved by the Ethics Committee of IFF.

## Results

**Fig 1:**
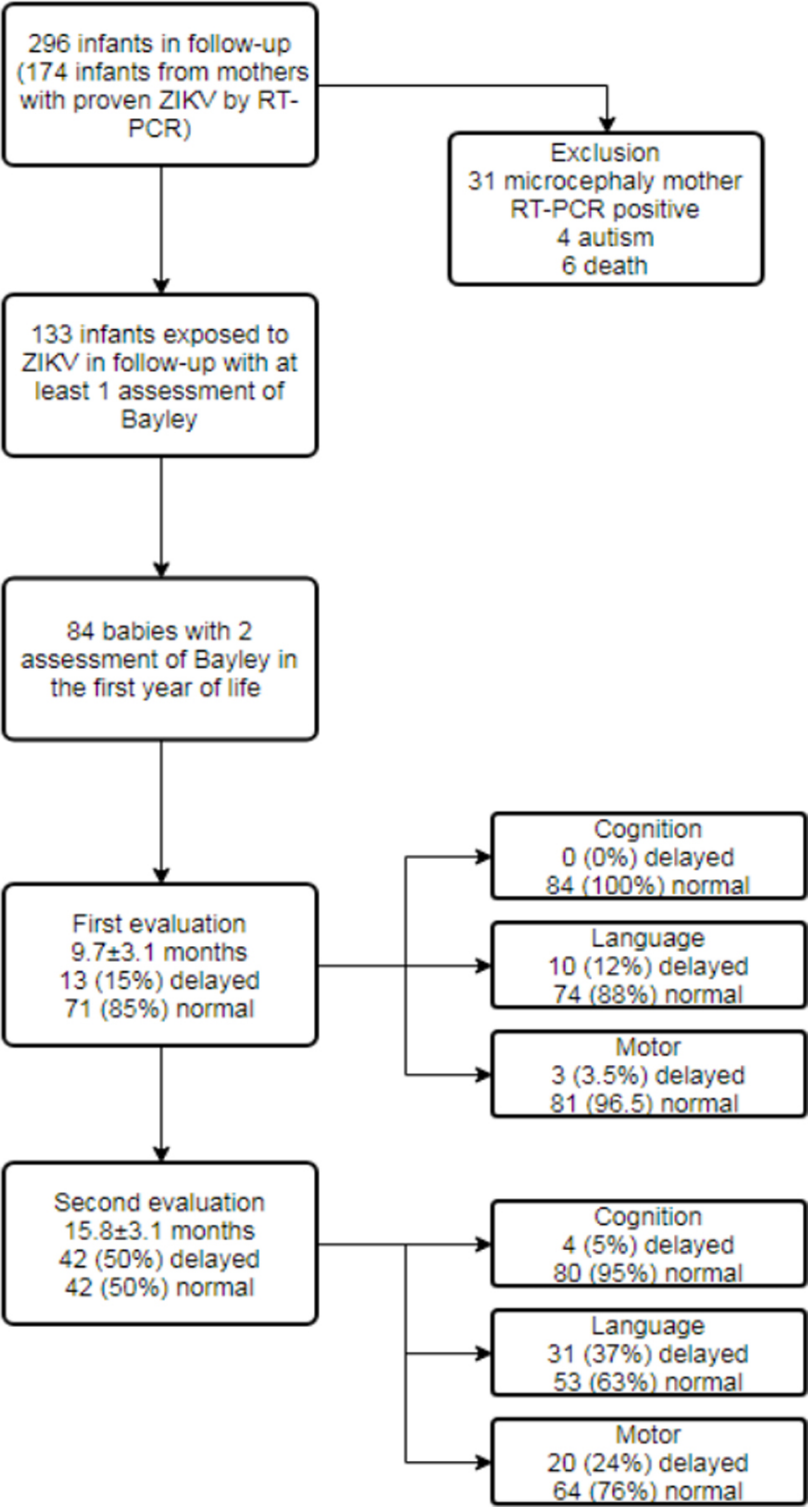
Flowchart of the study.

Two hundred ninety six infants with ZIKV exposure were followed at our institution as of May 2016 considering maternal symptoms and RT-PCR positive. Ninety-three infants presented CZS characterized by microcephaly, hydrocephaly, cerebral calcifications, cerebral atrophy or arthrogryposis. However, only 174 mothers of the infants included presented proven ZIKV with RT-PCR positive. Eighty-four asymptomatic infants from proven mothers exposed to ZIKV were enrolled for neurodevelopment evaluation using Bayley III Scales because they had at least two evaluations and their mothers had Zika RT-PCR positive. At birth their Dubowitz test, cerebral ultrasound and head circumference were normal (Fig 1).

At the time of the first Bayley III assessment, the mean chronological age of the children was 9.7±3.1 months (range 6–18 months). It was observed 13 children (15%) had a delay (considering score less then -1 SD) in one of the three domains, with following distribution: 10 (12%) in the language and 3 (3.5%) in the motor domain (Fig 1). None children had two or more domains affected simultaneously and 2 children had scores below 70 (-2 SD). At the time of the last Bayley III evaluation, the mean chronological age was 15.3±3.1 months (range 12–24 months). Forty-two children (50%) had a delay in one of the three domains: 4 (5%) in cognition, 31 (37%) in language, and 20 (24%) in motor performance. It was observed 7 children had at least two domains affected, 3 children had three domains affected and 8 children had scores below 70 (-2 SD) in at least one domain (Fig 1).

The maternal mean age at birth was 30.6±6.5 years (range: 16–42 years) and paternal mean age was 33.6±6.9 (range: 18–52 years). Almost half of mothers had a complete university education (n = 37–44%); most reported a household income higher than three minimum wages (n = 63–76%). No statistically significant difference were noted between demographic and income parameters in children with normal versus delayed development as the depicted in Table 1.

**Table 1:** Comparison of social demographic variables between normal and abnormal infant neurodevelopment.

Social demographics variables	Neurodevelopment		p-value
	Normal for age (n = 42)	Delayed for age (n = 42)	
Mother’s age (mean±SD)	31.9±7.1	29.9±5.9)	0.388
Father’s age (mean±SD)	33.3±7.3	33.9±6.6	0.704
Tertiary mother’s level of education,			
Yes (n %)	18 (42.9)	19 (45.2)	0.750
No (n %)	24 (57.1)	22 (52.3)	
No information (n %)	0 (0.0)	1 (2.4)	
Household income			
< 2 MW (n %)	10 (23.8)	9 (21.4)	0.656
From 3 to 5 MW (n %)	18 (42.9)	22 (52.4)	
≥ 6 MW (n %)	13 (31.0)	10 (23.8)	
No information (n %)	1 (2.4)	1 (2.4)	

SD: standard deviation MW: minimum wage at the moment of the evaluation (US$ 288.4/month [19])

For most mothers, infection occurred at the 2^nd^ trimester of gestation, with a low proportion reporting alcohol and tobacco use. All 84 children were tested by RT-PCR after birth. Between them, 17 were positive and 67 negative. There was a high proportion of cesarean section. None of the gestational variables or neonatal variables were significantly associated with developmental delay, as shown in Tables 2 and 3, respectively.

**Table 2:** Comparison of gestational variables between normal and abnormal infant neurodevelopment.

Gestational variables	Neurodevelopment		p-value
	Normal for age (n = 42)	Delayed for age (n = 42)	
Trimester of infection, n (%)			
1^st^ (n %)	8 (19.0)	8 (50.0)	0.639
2^nd^ (n %)	28 (66.7)	24 (46.2)	
3^rd^ (n %)	6 (14.3)	9 (60.0)	
No information (n %)	0 (0.0)	1 (2.4)	
Hypertension (n %)	8 (19.0)	3 (7.1)	0.097
Diabetes (n %)	3 (7.1)	2 (4.8)	0.500
Alcohol use (n %)	5 (11.9)	3 (7.1)	0.356
Tobacco use (n %)	2 (4.8)	2 (4.8)	0.692
Type of delivery			
Vaginal (n %)	12 (28.6)	8 (19.0)	0.306
Cesarean section (n %)	30 (71.4)	34 (81.0)	
Multiple gestation (n %)	2 (4.8)	5 (11.9)	0.216

**Table 3:** Comparison of neonatal variables between normal and abnormal infant neurodevelopment.

Neonatal variables	Neurodevelopment		p-value
	Normal for age (n = 42)	Delayed for age (n = 42)	
Gender			
Male (n %)	26 (61.9)	19 (45.2)	0.126
Female (n %)	16 (38.1)	23 (54.8)	
AP 5^th^ minute (mean±SD)	9.2±0.6	9.1±0.8	0.618
GA weeks (mean±SD)	38.6±1.6	38.2±2.0	0.265
BW g (mean±SD)	3,264.2±516.5	3,070.7±541.5	0.097
BW Z-score (mean±SD)	0.3±1.1	0.0±0.8	0.173
HC cm (mean±SD)	35.0±2.7	34.2±1,6	0.105
HC Z-score (mean±SD)	1.0±12	0.7±1.1	0.152
Classification			
SGA (n %)	4 (9.5)	2 (4.8)	0.498
AGA (n %)	33 (78.6)	37 (88.1)	
LGA (n %)	5 (11.9)	3 (7.1)	

AP–APGAR; GA–gestational age; BW–birth weight; HC–head circumference; SGA–small for gestational age; AGA–appropriate for gestational age; LGA–large for gestational age.

Exclusive breastfeeding was maintained in 49 (58.3%) children up to 6 months of age; hospital admission occurred for 9 (10.7%) children, and 56 children (66.7%) showed neurologic abnormalities during clinical follow-up (hypertonia n = 42, hypotonia n = 30, hyperreflexia n = 43, seizures n = 5, hyperexcitability n = 11, spasticity n = 2, irritability n = 9, tremors n = 3). Although the HC Z-score mean was within the normal range both at birth and at the time of the last examination, the difference between these measurements (Delta HC) was significantly lower in the developmentally delayed group, as seen in Table 4.

**Table 4:** Comparison of breastfeeding and clinical outcome variables between normal and abnormal infant neurodevelopment.

Breastfeeding and clinical outcome	Neurodevelopment		p-value
	Normal for age (n = 42)	Delayed for age (n = 42)	
Exclusive breastfeeding at 6 months	26 (61.9)	23 (54.8)	0.507
Hospital admissions	4 (9.5)	5(11.9)	0.500
Neurological abnormalities	24 (57.1)	32 (76.2)	0.064
HC at the last Bayley evaluation	47.4 (2.2)	47.7 (1.6)	0.387
HC Z-score at the last Bayley evaluation	0.9 (1.0)	0.7 (1.0)	0.458
Delta HC	-0.1 (1.3)	0.1 (1.1)	0.007

HC: head circumference; Delta Z-score HC: difference between Z-score HC mean at last examination and at birth.

## Discussion

Much is still unknown about the neurodevelopment of infants with congenitally acquired ZIKV infection who are asymptomatic at the time of birth[21–24]. We found progressive developmental delay in all functional domains using the Bayley III Scales assessment tools for cognition, language, and motor function from 6 to 24 months of age. In our cohort, the most affected component was language. These results agree with several studies that used animal models and suggested ZIKV infection could cause postnatal developmental deficit [25,26].

Language competence depends on the integration of many neural systems for adequate development, including cognitive development, hearing, central auditory processing, motor function, vision and visual information processing [27]. All these processes take place concomitantly with the evolution of cognitive, psycho-emotional and neuromotor development. Verbal performance is related to the maturation stage of the body’s motor development [28].

Our preliminary findings are of concern, for now there are yet no other comparative data. In vitro studies have demonstrated that ZIKV infects and damages human neural stem cells [29]. Cugola *et al*. demonstrated that Brazilian ZIKV strains cross the placenta and cause microcephaly by targeting cortical progenitor cells, inducing cell death by apoptosis and autophagy, and impairing neurodevelopment [30]. Additionally, our group showed that persistent ZIKV infection was found in the brain of a deceased five month old infant with CZS raising the hypotheses that brain damage might not be restricted to the antenatal period [31].

The role of infection during pregnancy in child development was already described in the classical group of teratogenic pathogenesis is referred to as TORCH [32]. A fetal infection may result in a systemic inflammatory response that can persist postnatally, compound injury to brain. This is one of the hypothesis to explain cerebral injury [32]. Additionally lesions indicative of deep gray matter injury, vascular compromise and neuroprogenitor cell dysfunction are describe [25,26,33].

Waldorf et al. inoculated ZIKV in pregnant primates and observed a loss of fetal neural progenitor cells, describing a characteristic pattern of cerebral injury in their fetus, even in the absence of microcephaly [34]. They identified a subtle injury pattern with the presence of a focus of periventricular hypersignal at T2, loss of non-cortical fetal cerebral volume, damage of the ependymal cells with gliosis, and loss of neural progenitor cell in subventricular and subgranular zones, with a dysmorphic neural granulation pattern. These modifications evidence the teratogenic effect of ZIKV, even in the absence of microcephaly, so the authors suggested the need for extended follow-up in the evaluation of neurodevelopment of children vertically infected with ZIKV [34].

The same recommendation was made by Saad *et al*. in a review of the most frequent clinical findings in children born to women with documented ZIKV infection, during gestation. They described a broad spectrum of abnormalities, which could be the result of an inflammatory reaction to the virus or a direct effect of the virus itself, causing CNS injury and neurological abnormalities [35].

One single marker for development delay which showed a statistically significant difference in our pediatric cohort was the delta HC. Different authors have reported the relationship between head circumference growth and neurodevelopmental abnormalities [36–39]. Raghuram et al. studied preterm newborns under 29 weeks of gestational age and showed that those with the worst head circumference growth had an increased risk for motor impairment and worst results in Bayley III Scales in all domains [40]. Neubauer et al. studied preterm newborns less than 32 weeks gestation age and verified that the HC at birth between 1 and 2 SD below the mean of the reference chart had no correlation with neurodevelopment at 12 and 24 months of adjusted age, but that delta HC below the mean at 3 months of age correlated with psychomotor delay [41]. As, a significant difference in the delta HC was observed, it is important to highlight that some investigator set al. consider this measure an essential marker to neurodevelopment, although it must be interpreted with caution due to the multifactorial nature of neurodevelopment [40]. Nevertheless, considering the known pathogenic effects of ZIKV on brain development and the lack of scientific knowledge regarding postnatal neurodevelopment of vertically infected children, delta HC should be a marker to be considered during the follow-up of ZIKV infected children.

Although asymptomatic at birth, our group of children developed a host of neurological abnormalities which demonstrated a statistical trend towards association with suboptimal.

### Limitations

A limitation of this study was that many of our families had difficulty maintaining appointments at regular windows, so for this reason we were not able to assess infants at exactly the same age. On the other hand, we were able to enroll a significant number of asymptomatic infants at birth, with documented ZIKV vertical exposure, who had at least two successive Bayley III evaluations in the first 24 months of age.

Other limitation is the absence of not exposed group in order to compare the influence of cofactors in neurodevelopment as maternal age (years), paternal age (years), maternal level of education and household income, that usually describe as related with development [42]. However, is not easy to determine who will be the not exposed group since more than 50% of the mothers could had asymptomatic disease during pregnancy and accurate serological tests are not available once cross reaction with Dengue virus are frequent.

Another issue that can be discussed is the cutoff point used to defined developmental delay (-1 SD) that can increase the frequency of abnormalities or describe only suboptimal neurodevelopment. However, this is the recommendation of the Bayley test manual and the study is been conducted in a pediatric hospital where wider cutoff points for developmental delays may be important for early referral for neuro-stimulation [16].

## Conclusion

Congenitally ZIKV-exposed infants who were asymptomatic at birth showed suboptimal neurodevelopment when evaluated by Bayley III assessments Abnormalities were mainly in the language domain during the first two years of life. The significant lower delta HC in the group with suboptimal development, as well as the concurrent presence of neurologic abnormalities are likely result of ZIKV infection in the developing brain.

Children with vertically ZIKV exposure require a longer follow-up period of evaluation in order for neurodevelopment to be adequately assessed. Studies in larger populations, evaluation of other confounding variables related to neurodevelopment and results of brain imaging studies should be taken into account for a better understanding of the broader effects of ZIKV on neurodevelopment.
